# Supplementary material for: Patterns of Intron Gain and Loss in Fungi
Source: PLoS Biol. 2004 Nov 30;2(12):e422. doi: 10.1371/journal.pbio.0020422 (PMC532390; doi:10.1371/journal.pbio.0020422)
Supplement: Table S1 — Also available at http://genes.mit.edu/NielsenEtAl/. (4.3 MB ZIP). [file pbio.0020422.st001.zip › NielsenEtAl/html/759.html]

AN4564.1.NCU00622.1.MG02875.1.FG08703.1


```
 CLUSTAL W (1.82) Multiple Sequence Alignments - Introns Inserted


Sequence 1: NCU00622.1	1491 aa
Sequence 2: MG02875.1	1504 aa
Sequence 3: FG08703.1	1468 aa
Sequence 4: AN4564.1	1474 aa
Alignment Length: 1567 aa
Number Identitical Residues: 522 aa
Alignment Score (without introns) 27153


MG02875.1 	MSFLFKSKKNQERSL-SSRDGPP-QSSNSSIQS-TAARVAREEKNALQRSTPTGSLH-SL
NCU00622.1	MAFLFKSKKNHDRGP-GSRDGIPGPGSASSTQS-ATARVRDEKG---SRSTPTGSLN-SL
FG08703.1 	MAFLFKSKKHPDKSQSGSRDAGS--GSQGSIQS-ITARAA-EKGSLQHRATPTGSVN-SI
AN4564.1  	MAFLFKSKKNQQTSGLPPATRNVHTSEGAPAGSGATLNGSKDGVVTSQTPTPSGSYNNSL
          	*:*******: : .   .       .. ..  *. : .   :       .**:** :.*:

MG02875.1 	DNEGSVSSPDQ-GYGR-----------RPQTAPNEQGLG--~--TPPSDLP0FRNGGQPQ
NCU00622.1	DNDGSVGSPEQQNYGRPRGQTLEGQHQHQQQAPSQMQSREP~PQQPPNDLP0FRNGANPN
FG08703.1 	ENEATSGSPDQ-GPGP----------ALSHGHGRRGGSADQ~STQQPSDPS0LRNGPP--
AN4564.1  	NSVTSTNSPEQ------------------QRMRQRAESESQ0AQRPQQSTS~P-------
          	:.  : .**:*                  :    .    .  .    .. .         

MG02875.1 	PAPQQVSSPNSNLYPWTQRRLNYTTSHPSPFPRYGAAVNSTSSKEGDIYVMGGLINSATV
NCU00622.1	PMP--MANPNASLYPWSQRRLTYTTSQPSPFPRYGAAVNSVSSKEGDIYVMGGLINSSMV
FG08703.1 	-----LNGPNASLYPWSQRRLTYTSTHPPPFPRYGAAVNSVSSKEGDVYLMGGLINGSTV
AN4564.1  	-----VSSPGSSLYPWSQRRLNFSSPQANPFPRYGAAINAVASKEGDIYMMGGLIDGSTV
          	     : .*.:.****:****.:::.:. ********:*:.:*****:*:*****:.: *

MG02875.1 	KGDLWMIEAG-GNLSCYPLSTTAEGPGPRVGHASLLVGNAFIVYGGDTKIDEADVLDETL
NCU00622.1	RGDLWMIEAG-GSMACYPLATTAEGPGPRVGHASLLVGNAFIVFGGDTKIDESDVLDETL
FG08703.1 	KGDLWMIEAG-GNMACYPLATTAEGPGPRVGHSSLLVGNAFIVYGGDTKIDESDVLDETL
AN4564.1  	KGDLWMLESSSGNLSCFPIATVSEGPGPRVGHASLLVGNAFIVFGGDTKVDENDTLDDTL
          	:*****:*:.:*.::*:*::*.:*********:**********:*****:** *.**:**

MG02875.1 	YLLNTS1TRQWSRSLPAGPRPSGRYGHSLNILGSKIYIFGGQVEGYFMNDLAAFDLNQLQ
NCU00622.1	YLLNTS1TRQWSRALPGGPRPAGRYGHTLNILGSKIYVFGGQVEGYFMNDLAAFDLNQLQ
FG08703.1 	YLLNTS1TRQWSRALPSGPRPSGRYGHSLNILGSKIYVFGGQVEGLFMNDLSAFDLNQLQ
AN4564.1  	YLLNTS1SRQWSRSIPPGPRPTGRYGHTLNILGSRLYVFGGQVEGYFFNDLVAFDLNQLQ
          	****** :*****::* ****:*****:******::*:******* *:*** ********

MG02875.1 	MQDNRWEMLLQNSDSGGPPVGTVPPARTNHTMITYNDKMYL2FGGTNGFQWFNDVWCYDP
NCU00622.1	MPSNRWEMLIATTEPGTTPQGNVPPARTNHSMVTFNDKMYL2FGGTNGFQWFNDVWCYDP
FG08703.1 	MPNNRWEILVHGETSP-----KMPAARTNHTMITFNDKMYL2FGGTNGFQWFNDVWCYDP
AN4564.1  	NPGNKWEFLIQNSHEGGPPPGQIPPARTNHTIVSFNDKLYL2FGGTNGLQWFNDVWSYDP
          	  .*:**:*:       .. . :*.*****:::::***:** ******:*******.***

MG02875.1 	ASNSWSQLDCIGYIPIPREGHAASLVDDVMYIFGGRTEEGADLGDLAAFRITSRRWYTFQ
NCU00622.1	STNAWTMLECIGYIPVPREGHAAAIVDDVMYIFGGRTEEGADLGDLAAFRITSRRWYTFQ
FG08703.1 	AVNKWSQFDCIGYIPAPREGHAAALVDDVMYVFGGRTEEGTDLGDLAAFRISSRRWYTFQ
AN4564.1  	RTNSWTQLDCVGFIPTPREGHAAALVNDVMYVFGGRTDEGIDLGDLAAFRISTRRWYSFQ
          	  * *: ::*:*:** *******::*:****:*****:** **********::****:**

MG02875.1 	NMGPSPSPRSGHSMTTVGKAVVVVGGEPSTAATAVNDLGLIYMLDTTKIRYPNDS-QIQA
NCU00622.1	NMGPSPSPRSGHSMTAVGKTVVVVGGEPSSATAAVNDLALVYCLDTTKIRYPPDS-AGNP
FG08703.1 	NMGPSPSPRSGHSMTTVGKSIVVLGGEPSSATASVSDLGLLYVLDTSKIRYPNDAPQTSQ
AN4564.1  	NMGPAPSPRSGHSMTAFGRQIIVLAGEPSSAPRDPVELSMAYMLDTSKIRYPTEN-PNGE
          	****:**********:.*: ::*:.****:*.    :*.: * ***:***** :      

MG02875.1 	NQRQQGTRRPSTSNSNEAGGMQVANRALPLREGSQGPPE-KRMMAPREAGAVQSPPNGVR
NCU00622.1	GAPRNAVRRPSNATPPSS------SQNFPPRDGSSGPPDQRRLVGPPGDAGNTTPQNGFR
FG08703.1 	PPRVQGSRRPSASE---------GNRPYPARDGSNGPSDSRKMIVG----GPSAPPNGHR
AN4564.1  	KAMFPGPTKTGAGEKPPSGRVSREAQNQPSDQFRRAQGPRESVVSPTGRPTELGPGPGSR
          	     .  :.. .    :.  .   :  *  :   .    . ::          *  * *

MG02875.1 	GP---------NGTTDASKLPRAAGAP--PNNTQPQG-PPQKPAVNTVAAPRSRNTSRDR
NCU00622.1	GPPNPNDQIGPRGPPNGPPKPMRPGPPMGPAGPPPQG-QPPRPGVNPAVARARNASSDSR
FG08703.1 	SPPNSVEVEGSQPAIANAKLPRASAMP--PSGPPPQGPMPARPTVDVSAVARVRGQSAER
AN4564.1  	LPRAS-----IANAPAGPPPPGQAPTP-GPRGNPPRDGNNFRSKTPTKQERGYGGPPIDT
          	 *  .        .   .  *  .  * .* .  *:.    :. .           . . 

MG02875.1 	ADMVVSP--VTPIQNASPVIPDNTKMVDLPTN-GRRTPTSQGAGPKAATAPKLEP-VVET
NCU00622.1	ADSYQGSPTQSPIVQQGPVFTQNE---SSGSV-GRHTPTQNAPRSASSASRQTEPPAAET
FG08703.1 	AGASGSP---RTMHSGSPITREVISEVDSPASNGQRTPVQQPPR----VASRQDQVSGDV
AN4564.1  	TRAIAGDREREPAPRDSPKDPRFA-----QESTGQRTPTQQSQRMSARAMEAGEAAPLVA
          	:    .     .    .*              .*::**..:     : .    :     .

MG02875.1 	PKPAQARSIKSQGSVDSSTDPGLK-AAAVRAASPPPP-TRQNSN-PNRRSSNRNSQTVVL
NCU00622.1	PKSKPLKQARTQGSVDSSTDANLK-QAANRSASPPAPTARLNSNSINRRSSQRNSQTVVL
FG08703.1 	SKIKQAPQGRPQGPADGAPEASIKPTIAPRPASPPVP-ARQPSNNINRRSSGRNSQTVVL
AN4564.1  	PARQRSLRQRQRSSMDSADESILG-RHASIDGSVDSRTHRNSRTGDEPRSPRLTAHQEAL
          	.        : :.. *.: :. :    *   .*    : *   .. : **.  .::  .*

MG02875.1 	LKELDMARNRNAWYASELELARKAGYTPNASMSPVFERGAAETFDDEDKPLIEALLAMRQ
NCU00622.1	LKELDAARNRNAWYASELELARKAGYVPTASMSPALDQRAAETFDDEDRPLIEALLAMRT
FG08703.1 	LKELDSARNRNAWYASELELARKAGYVPNTSYSPLLDNKATETFDDEDRPLIEALLAMRS
AN4564.1  	FKELEALKSRNAWYASELALAKKAGYTPNISANSALDERTSDAFTDEDRPLIEAFLAMRA
          	:***:  :.********* **:****.*. * .. ::. ::::* ***:*****:**** 

MG02875.1 	ELANVQISVDKQAVLAAKQIAEAEKQRDAAIQEAVYAKARLAGQVGGSLSSTPQMD----
NCU00622.1	ELANVQNSVDKQAVLAAKQIAEAERQRDAAIQEAIYAKAKLAAQ-GGSARSTPQLD----
FG08703.1 	ELANVQTAVDKQAVVAAKQIAEAEKQRDAAIQEAIYSKAKLAAHLGGSSASTPQLD----
AN4564.1  	ELAKMQATVDRQAAIASKRVAEVEQQRDVAVNEAAYARAKLAAH-GGSQQSTPSLDGQSH
          	***::* :**:**.:*:*::**.*:***.*::** *::*:**.: ***  ***.:*..: 

MG02875.1 	-LARDPDD-RDGELSRKLAATLSSQKDLQSQVDRMSAELEAEKRARQLADDTANAAQKRM
NCU00622.1	-MDKDGND-RVGEMSRKLAQALSIQKDLQDQVDRLKIDLDAEKKARKLADDTASAAQKRM
FG08703.1 	-GPKDDSELRSVELSRKLASALHVQKDLQSRLDSARSELDSERKARLLADDTSNAAQKRM
AN4564.1  	DLDKAASD-RTTDLSRRLALALASQNELKSKLEALSTDLEQEKRGRELAEETSEATRRRL
          	.  :  .: *  ::**:** :*  *::*:.:::    :*: *::.* **::*:.*:::*:

MG02875.1 	RDLETFKQQNSLETESLRAELHILQRESREQSIAAAEAQAQTDTLRVEKEELDAKLKELT
NCU00622.1	ADLEHYKQKNSGEVEQLKAEMHMLQRQAREAEVAASEFESTAQMLRLEKDELENKYNEAV
FG08703.1 	AELESYKQQTSTEVERLKAELHLAQHEAREHSVARAEVAATVELLHIEKNDIEQKYHEAI
AN4564.1  	AELEMQN--NPLETESLRAQLHQVEASFREESMLRSEAESSLKQLALDKEELSRKLEDST
          	 :**  :  .. *.* *:*::*  : . ** .:  :*  :  . * ::*:::. * .:  

MG02875.1 	GNSEESSNALTGLRKALAASTEIRELLERKAEEERANREEIEAKLIRLKAEHEQRTAELE
NCU00622.1	GNSKQSVETFQSLREAVAATTEMRETLERKLDEERALREEVEATLNKLKAEREVQAAELA
FG08703.1 	GNSKDHSEAFDSLRAAIVASEDSTALLENKLAEERSQREKIETQLNKLKFEHEARTAELV
AN4564.1  	NRLRDFGDNIGGLKQAVTASVEKASILEKKLDEERERREGLERKLLQLRSEHEERTAELE
          	.. .:  : : .*: *:.*: :    **.*  ***  ** :*  * :*: *:* ::*** 

MG02875.1 	AATARLRDAEQLAERHANEAKAHKQALISGLDQITARSVSKSGKGDSD-RIVALQGQLNA
NCU00622.1	TITQRLRDAEERAERHANEARVHRQAVLAGLDKLPTMENRNLGNGEID-RTSALQGQLSA
FG08703.1 	ATTQRLRDAEQLSEKHATEARTTRQAVLAGLDKISARDISGPSKADGE-RITALQTQLAT
AN4564.1  	NATRRLRDAEELAESHAREAEAHKNAFILGLERASSFDSETSIRSLVDQRVATSEAQVER
          	  * ******: :* ** **.. ::*.: **:: .: .     ..  :.*  : : *:  

MG02875.1 	ANAMVRKYQEQADTASDKLRSAEERIAGLEAYQEQASRDGVHIRKQLQAAMRDTQSLQAE
NCU00622.1	ANALVRKYQQEADSASDKLRIAEERIAGLEAYQEQASREGVSIRRQLQSALRETQSLQAA
FG08703.1 	SKELVKKYQQEVEAATDKLRGAEERIAGLEQYQEQSSREGVTIRRQLQSALRDTQSLQAA
AN4564.1  	ANKLARASQAAADEAAEKLRRAEERIAGLEAYQEQASREGLQLRRQLQAALKDCQTYTAE
          	:: :.:  *  .: *::*** ********* ****:**:*: :*:***:*::: *:  * 

MG02875.1 	NSDLKSQLSAQQLETNAITVQHNTLKDILSERGISPNGAPRSR--ASPRGETPDTA--AA
NCU00622.1	NSDLKQQLAKQQLETNAVLVQHNTLKDILTERGISPTSVNRHSRLGSPREGSPE----AM
FG08703.1 	NTDLKNQLAAQQLETNAMTVQHNALKDILSERGISPTASIRTRGITNPRINSPAISPDLN
AN4564.1  	NRELKAQIENHQREAGALAVQHAALKDLLGERGYTDSRRSPRGESPGSRFGTPE----QN
          	* :** *:  :* *:.*: *** :***:* *** : .         ..*  :*       

MG02875.1 	RVRELEQQLQAAQQAQEEHRQAFMTQAQGTESAYREKLSQLENDYQSAVHYVKGTEKMLK
NCU00622.1	RLRELEQQLAAAQAAHEETKASAAKQAQEAETTYRDKLAQLDNDYQSAVHYVKGTEKMLK
FG08703.1 	RVRDLESQLASSSAAHEETKQVFAIQVQESEAAWREKLSQLESDYQSAVHYVKGTEKMLK
AN4564.1  	RLRELEQQLSASLKAHDELKASFETREQETDRAYKEKLEQLENDYQSAVHYVKGTEKMLK
          	*:*:**.** ::  *::* :     : * :: ::::** **:.*****************

MG02875.1 	KLKEELARYKNENSRLKSQIGDMEDTKG--PSITNTPAEWESERATLTKKIETLQSEMMT
NCU00622.1	QLKEQLTRYKTENSRLKEQIVELEDKVGGETGGTGAPADWETERESLQGQVSELQAELQR
FG08703.1 	QLKDQLSRYKTENARLKTEIEDLEDNAQ--AGASSTSANWESEKAELQARIRSLETELEN
AN4564.1  	RMKDELTRYKMQNAKIQSELEAAQSREG-----SVAPSGWEAERSQLQQSLTDLQQDTSR
          	::*::*:*** :*:::: ::   :.        : :.: **:*:  *   :  *: :   

MG02875.1 	ATTQLEKQMQAVRTELEAAKRERDNAVKHSEEASQRVNTN----KKDLEQLQQENSLLEQ
NCU00622.1	TASQLDQQLKSLREELEDVRQERDSAAHASDDASRRLEAT----KKDLEELQHENALLER
FG08703.1 	TAAQMEKRLAGLQTELQATS-------QHHEAATQKLNSH----RKDLEQLQSENALLEQ
AN4564.1  	SITNLEEQITSLKESLASAEAEKEKSVAEYETMRQELLAVSEKTSSELEQLKHENSLLEA
          	: ::::::: .:: .*  .  . ..:    :   :.: : :..: .:**:*: **:*** 

MG02875.1 	RAQDAEKKVNLLLDQVESSVDNYRRRSRQVASMNSDQLRESLASMEAAEAKSAAAAKTNG
NCU00622.1	RARDAEQKVSTLLDQVEMSVDNYRRRSRQVP----------------NEAIGAAVTP---
FG08703.1 	RANDAEQKVALLLDQVEHSVDNYRRRSRQVPSVN------------SEVAVGANGMG---
AN4564.1  	RAMDAEQKVSMLLDQVEASVGHYRRQSQHGQGVN---------------GISRTHSN---
          	** ***:**  ****** **.:***:*::  . .               . .        

MG02875.1 	KLGHSTGASDSVSTEGSTGGGLSGLGGHLRSDSLDTTSEYNSG-GERNSAALDNLANELE
NCU00622.1	-----VGPSNEIG--------------HVRHESSEAESLYGPGPDARDSVALDHLTSELE
FG08703.1 	-----LGHSRQESSEGE-----SVYGSANGNGNGSGNGNGNGGLEARNSAALDSLANELE
AN4564.1  	------ASSGTIG------------AGTRRSRANSAVSQDDTFLDNRGSMALDSLANELE
          	      . *   .            ..       .  .  .     *.* *** *:.***

MG02875.1 	TLRTHWEATNKNYRLSNTFDFEGSGMPTPTAATSGAGGGGPGGKKAGDKDDKGLSESLAD
NCU00622.1	TLRSRWETNSKNYRHSNAFDFEGLGTPTASLPNQKSDGTLGLSESMADWRKKNFDDSHLD
FG08703.1 	TLRSQWEATNKNYRLSTNFDFDAS---SGVKKEGDVTAGVGLSESLADWRKRLDTEETHN
AN4564.1  	ALRSHWESTNSNYRLSTQSDFDRTPTKDSGLSDSLAEWRRRLDEEEARAGSPEKGKPRTA
          	:**::**:...*** *.  **:                    .:. .   .    .    

MG02875.1 	WRKRLDNA---
NCU00622.1	DDEHPPAGSAR
FG08703.1 	DGTSLDTSNRK
AN4564.1  	AEGQAAANMI-
          	
```
